# A Case of Fatal Gastrointestinal Anthrax in North Eastern Iran

**DOI:** 10.1155/2015/875829

**Published:** 2015-03-31

**Authors:** Seyed Ahmad Hashemi, Amir Azimian, Sara Nojumi, Tahereh Garivani, Saghar Safamanesh, Majid Ghafouri

**Affiliations:** ^1^Department of Infectious Diseases, Emam Reza Hospital, North Khorasan University of Medical Sciences, Bojnurd 74877-94149, Iran; ^2^Department of Pathobiology and Anatomy, School of Medicine, North Khorasan University of Medical Sciences, Bojnurd 74877-94149, Iran; ^3^Medical Diagnostic Laboratory, Emam Reza Hospital, North Khorasan University of Medical Sciences, Bojnurd 74877-94149, Iran

## Abstract

*Background. Bacillus* species are aerobic or facultative anaerobic, gram-positive, or gram-variable spore-forming rods. They are ubiquitous in the environmental sources. *Bacillus anthracis* may usually cause three forms of anthrax: inhalation, gastrointestinal, and cutaneous. The gastrointestinal (GI) anthrax develops after eating contaminated meat. In this paper we report septic intestinal anthrax. *Case Presentation*. We report an isolation of *Bacillus anthracis* from blood culture of patient with intestinal anthrax. *Bacillus anthracis* was isolated from a blood culture of a 34-year-old man who had a history of severe abdominal pain, bloody diarrhea, nausea, vomiting, fever, sweating, and lethargy within 4 to 5 days after eating the meat of domestic goat. He had evidence of severe infection and septic shock and did not respond to treatments and subsequently expired 9 hours after hospitalization. *Conclusion*. Gastrointestinal anthrax is characterized by rapid onset, fever, and septicemia. Rapid diagnosis and prompt initiation of antibiotic therapy can help in survival. Most of previous cases of septicemic anthrax were related to injection drug users but, in our case, septicemia occurred after gastrointestinal anthrax.

## 1. Introduction

*Bacillus* species are aerobic or facultative anaerobic, gram-positive, or gram-variable spore-forming rods. They are ubiquitous in the environment and are found in water, dirt, air, stools, and plant surfaces.

Anthrax is caused by* Bacillus anthracis*. It occurs when* Bacillus anthracis*' endospores enter the body either through breaks in the skin, ingestion, or inhalation. Anthrax characterization is based upon its original mode of transmission: cutaneous, gastrointestinal, and inhalational [1]. The incubation period for the gastrointestinal form is usually 2 to 5 days but may be as short as 15 hours. The gastrointestinal (GI) anthrax develops after eating contaminated meat. Because anthrax is more common in the developing world where reporting is not common, the true incidence of GI anthrax is unknown. There are some reports of gastrointestinal anthrax in different cities of Iran. GI anthrax has been reported in Mazandaran, Shiraz, Kermanshah, Rasht, and Mashhad [2–6]. All of these cases were related to sheep and goat meat.

We present an interesting case of septicemic anthrax, in a patient with history of eating goat meat and severe gastrointestinal disorders, in Bojnurd, Iran.

## 2. Case Presentation

*Bacillus anthracis* was isolated from a blood culture of a 34-year-old man who had a history of severe abdominal pain, bloody diarrhea, nausea, vomiting, fever, sweating, and lethargy within 4 to 5 days after eating the meat of domestic goat. He had evidence of severe infection and septic shock and did not respond to treatments and subsequently expired 9 hours after hospitalization. Physical examination on his admission was as follows: temperature = 36°C, pulse rate = 109 beats/min, blood pressure = 80/60 mmHg, and respiratory rate = 20 breaths/min. At the time of hospitalization urine, stool, and blood samples of patient were sent to a laboratory. In sonography, ileus in intestinal loop and edema of intestine with presence of free fluid were observed.

Laboratory studies were as follows: hemoglobin concentration 17.8 g/dL; hematocrit 50.6%; leukocytes 18400/mm^3^; and platelets 169000/mm^3^. In the urine analysis of the patient, urine was orange, semiclear with positive reaction for protein (trace), and sugary (one plus) and has 10–12 WBC per mL. Urine culture was negative. Stool sample was bloody but culture was negative for routine pathogenic bacteria. Biochemical tests showed that urea was 64 mg/dL, creatinine was 1.3 mg/dL, calcium was 7 mg/dL, blood sugar was 127 mg/dL, magnesium was 2.5 mmol/L, sodium was 138 mmol/L, and potassium was 3.5 mmol/L. Interestingly, two days after the patient's death, his blood culture showed growth of bacteria. Performing primary phenotypic tests indicated that the isolated bacterium was* Bacillus anthracis*. Gram-staining microscopy showed nonbranching gram-positive bacilli, grown in chains. Subcultures represented typical growth of aerobic spore-forming bacilli without hemolysis. It should be noted that we do not have any sample of eaten meat or patient's environment. To maintain safety in our laboratory and also due to death of patient, we did not perform antibiogram tests and, under the terms of our Center of Disease Control and Prevention for final confirmation, we sent sample to Pasteur Institute, Iran branch (reference anthrax laboratory in Iran). After phenotypic and genotypic evaluations, reference laboratory confirmed our identification. In genotypic tests all three chromosomal markers,* pag* (pXO1),* capB* (pXO2), and* rpoB,* were detected by the PCR.

## 3. Discussion

In our study we present gastrointestinal and septicemic anthrax in young male who had eaten infected goat meat. Usually, the gastrointestinal (GI) anthrax develops after eating contaminated meat. When spores germinate in the intestinal tract, they cause ulcerative lesions. These lesions can occur anywhere and may, in severe cases, result in hemorrhage, obstruction, or perforation [7, 8]. Because of the propensity of* B. anthracis* infection of the gastrointestinal tract to progress to sepsis or death, both types of gastrointestinal anthrax should be treated aggressively as a systemic illness. Anthrax can be successfully treated if the disease is promptly recognized and appropriate therapy is initiated early (surgery and antibiotic therapy). Penicillin G (24.000.000 IU/day, IV) is the best choice for therapy.

The diagnosis of GI anthrax requires a high index of suspicion which may be diagnosed on the basis of epidemiology or microbiologic, pathologic, or serologic testing. Laboratory diagnosis may be obtained from stool, blood or peritoneal cultures, tissue samples, or serologic evidence of infection [2].

We presume that the source of* B. anthracis* in bloodstream of our patient was perforation in gastrointestinal tract. This is the fourth* B. anthracis* case in the last three months in our laboratory (Emam Reza Hospital, Bojnurd, North Khorasan, Iran), although it should be noted that the other cases of anthrax had not been found in the patient's family. Three previous cases were cutaneous anthrax. Totally, because of conventional dairy boom in rural areas of North Khorasan the relatively high prevalence of zoonotic infections such as anthrax is presumable and medical staff should be aware of that fact.
